# An Improved Enzyme-Linked Immunosorbent Assay (ELISA) Based Protocol Using Seeds for Detection of Five Major Peanut Allergens Ara h 1, Ara h 2, Ara h 3, Ara h 6, and Ara h 8

**DOI:** 10.3389/fnut.2019.00068

**Published:** 2019-06-05

**Authors:** Arun K. Pandey, Rajeev K. Varshney, Hari K. Sudini, Manish K. Pandey

**Affiliations:** International Crops Research Institute for the Semi-Arid Tropics (ICRISAT), Hyderabad, India

**Keywords:** protocol, peanut allergens, Ara h 1, Ara h 2, Ara h 3, Ara h 6, Ara h 8, ELISA

## Abstract

Peanut allergy is an important health concern among many individuals. As there is no effective treatment to peanut allergy, continuous monitoring of peanut-based products, and their sources is essential. Precise detection of peanut allergens is key for identification and development of improved peanut varieties with minimum or no allergens in addition to estimating the levels in peanut-based products available in food chain. The antibody based ELISA protocol along with sample preparation was standardized for Ara h 1, Ara h 2, Ara h 3, Ara h 6, and Ara h 8 to estimate their quantities in peanut seeds. Three different dilutions were optimized to precisely quantify target allergen proteins in peanut seeds such as Ara h 1 (1/1,000, 1/2,000, and 1/4,000), Ara h 2 and Ara h 3 (1/5,000, 1/10,000, and 1/20,000), Ara h 6 (1/40,000, 1/80,000, and 1/1,60,000), and Ara h 8 (1/10, 1/20, and 1/40). These dilutions were finalized for each allergen based on the accuracy of detection by achieving <20% coefficient of variation in three technical replicates. This protocol captured wide variation of allergen proteins in selected peanut genotypes for Ara h 1 (77–46,106 μg/g), Ara h 2 (265–5,426 μg/g), Ara h 3 (382–12,676 μg/g), Ara h 6 (949–43,375 μg/g), and Ara h 8 (0.385–6 μg/g). The assay is sensitive and reliable in precise detection of five major peanut allergens in seeds. Deployment of such protocol allows screening of large scale germplasm and breeding lines while developing peanut varieties with minimum allergenicity to ensure food safety.

## Introduction

Food allergies have become the most important food safety concerns around the world. The prevalence of food allergy is more in North America, where 6% of young children and 4% of adults suffer from food allergies (1). Among all the food allergies, ~1.4% of Americans and 3% Australian are allergic to peanuts and peanut-based products (2, 3). Peanut allergy is very severe and sometimes causes death due to anaphylaxis (4, 5). It is reported that food allergies cause approximately 150 to 200 fatalities per year, based on data from a five year study of anaphylaxis in Minnesota from the Mayo Clinic. Fatal food anaphylaxis is most often caused by peanuts (50–62%) and tree nuts (15–30%) (6). Proteins are the major cause of food allergy and these allergenic proteins are usually highly resistant to heat and proteolysis (7). Peanut is the largest source for the IgE-mediated food allergies among eight major sources namely tree nuts, egg, cow's milk, fish, crustacean shellfish, soybean, and wheat. There is no effective treatment to peanut allergy and the allergic person is advised strictly to avoid consuming peanut or peanut-based products (8). Peanut being a common food ingredient in many food preparations, it is very challenging for the allergic person to know the composition of these preparations to avoid consumption (9). The threshold of allergen levels differ among the allergic population and even a minute dose of 100 μg of Ara h 1 can trigger an allergic reaction (10). The diagnosis of peanut allergy can be done using different methods such as double-blind, placebo-controlled food challenge (DBPCFC), the basophil activation test, the specific skin prick test (SPT), and the measurement of specific IgE (11–13).

Of the 32 different types of proteins present in peanut seeds, 16 of these proteins show allergenic property (14). Further, out of these 16 peanut allergen proteins, Ara h 1, 2, 3, and 6 are considered as major allergens because they are recognized by the IgE of a majority of allergic patients. Ara h 5, 7, 8, 9, 10, 11, and 12/13 are considered minor allergens and are not thought to be the causative agents in most of the life-threatening allergic reactions (anaphylaxis) (15). Nevertheless, if a person is already sensitive to Bet v 1 allergey caused due to birch pollen, then one of these minor peanut allergens, Ara h 8, shows cross-reactivity with IgE antibodies causing oral allergy syndrome (OAS) (16–18).

Allergic protein belongs to different protein families namely cupin (vicilin-type, 7S globulin, legumin-type, 11S globulin, glycinin), conglutin (2S albumin), profilin, nonspecific lipid-transfer protein 1, pathogenesis-related protein, PR-10, 14 kDa oleosin, and 16 kDa oleosin (19), particularly, Ara h 1, Ara h 2, Ara h 3, and Ara h 6 are the seed storage proteins. Many studies showed that Ara h 1 and Ara h 3 are abundant peanut proteins but, Ara h 2 and Ara h 6 strongly bind with IgE from peanut allergic patient and release mediators from basophils, which confirmed that both are the most important allergens *in vitro* (20–23) and *in vivo* (21, 24, 25) with regards to food allergy (23). Due to the fact that both Ara h 2 and Ara h 6 are recognized more prominently and more frequently than Ara h 1 and Ara h 3 by peanut allergic children with almost all of them showing IgE reactivity to these two allergens (26).

Immuno-assays are the most commonly preferred analytical techniques being used to detect the food allergens in many industries (27). Food industries commonly use ELISA to detect and quantify hidden allergens in food commodities as it is easy and less time-consuming technique (28–30). Further ELISA is highly sensitive, reliable, cost-effective, and accurate. In the past ELISA has been successfully used to detect allergens in several food products such as in meat and meat products, milk and milk products, fish and fish products, soybean, nuts and nut-based products, and fruit juices and their ingredients (31, 32). In ELISA, allergens can be detected by the colorimetric reaction after binding of the antigen with the specific enzyme-labeled antibody. Earlier studies reported the allergenic proteins quantification in peanut-based products through ELISA and HPLC technique (29, 33–35), however, no information on peanut seeds. The key issues in ELISA are the sample preparation and unacceptable variability in multiple measurements of the same sample. The best way of preparing a sample, right dilution of the sample used in the assay, and low coefficient of variation (CV) holds importance in achieving accuracy. As of now there is no standard protocol which can help to detect allergen proteins in peanut seeds on the basis of dilution factor and low coefficient of variation (CV). Further the genetic diversity available in peanut germplasm has not yet been fully exploited and the accumulation of favorable alleles may provide minimum allergen proteins in the improved peanut genotypes. At this juncture a highly sensitive, easy to use and reliable protocol for peanut seeds is required to screen various germplasm sets and breeding material. Therefore, the present study focused on developing a robust protocol to estimate major allergens in peanut seeds through ELISA based methods.

## Materials and Methods

### Plant Material, Reagents and Chemicals

A total of 38 peanut genotypes (see Supplementary Table 1) were used while improving and standardizing the ELISA protocol. The seeds of these genotypes after harvest were stored at 10°C and were taken out only when needed for the experiment. ELISA components such as peanut allergen standards, monoclonal antibodies (MAbs; primary and secondary biotinylated antibody), and enzyme conjugates were purchased from INDOOR Biotechnologies Inc. (Charlottesville, VA, USA) for the estimation of five important peanut allergens namely Ara h 1, Ara h 2 Ara h 3, Ara h 6, and Ara h 8. These include monoclonal antibody 2C12 (clone 2C12 A11 A3) and biotinylated antibody 2F7 (clone 2F7 C12 D10) for Ara h 1, the monoclonal antibody 1E8 and biotinylated monoclonal antibody 4G9 for Ara h 3 while monoclonal antibody 3B8 (clone 3B8 B5) and biotinylated antibody 3E12 (clone 3E12 C4 B3) for Ara h 6. Similarly, the monoclonal antibody 1C4 (clone 1C4 G4 C8) and a polyclonal rabbit anti Ara h 2 antibody (AH2) for Ara h 2; capture antibody 4G6 and a polyclonal antiserum raised in rabbit for Ara h 8 were purchased from INDOOR Biotechnologies Inc. Conjugated Goat anti-Rabbit IgG was purchased from Jacksons Laboratories (Bar Harbor, USA Cat No. 111-036-046). ABTS™ (Cat No. 11204521001) and all other reagents and chemicals were purchased from Sigma-Aldrich Co. (Oakville, ON, Canada).

### Allergen Standards and Enzyme Solution Preparation

The allergen standards were purchased from INDOOR Biotechnologies Inc. (Charlottesville, VA, USA). These standards were isolated from lightly roasted peanut flour (Runner cultivar) and purified by affinity chromatography. The purified standards were supplied in phosphate buffer, received on the ice and stored at −20°C until further use. Purified natural Ara h 1 (Lot 39285; Conc. 20,000 ng/ml), Ara h 2 (Lot 39158; Conc. 2,500 ng/ml), Ara h 3 (Lot 39051; Conc. 1,250 ng/ml) Ara h 6 (Lot 39198; Conc. 1,000 ng/ml), and Ara h 8 (Lot 39033; Conc. 2,500 ng/ml) were used as allergen standards for each assay. The standard concentration of each allergen ranged from 2000 - 4 ng/ml for Ara h 1, 250 - 0.5 ng/ml for Ara h 2, 125 - 0.24 ng/ml for Ara h 3, 100 - 0.2 ng/ml for Ara h 6 and 250 - 0.49 ng/ml for Ara h 8. ABTS Substrate is a water-soluble peroxidase substrate that yields a measurable green end product for use in ELISA methods. The ABTS™ (Sigma Aldrich Cat No. 11204521001) was dissolved in 1 mM ABTS solution. The ABTS solution contains 0.1M anhydrous citric acid and 0.2M Dibasic Sodium phosphate.7H_2_0_2._ The 274 mg ABTS™ were dissolved in 500 ml ABTS solution and store in an amber color bottle at 4°C until use.

### Development of Protocol for Allergen Estimation

**Figure 1:**
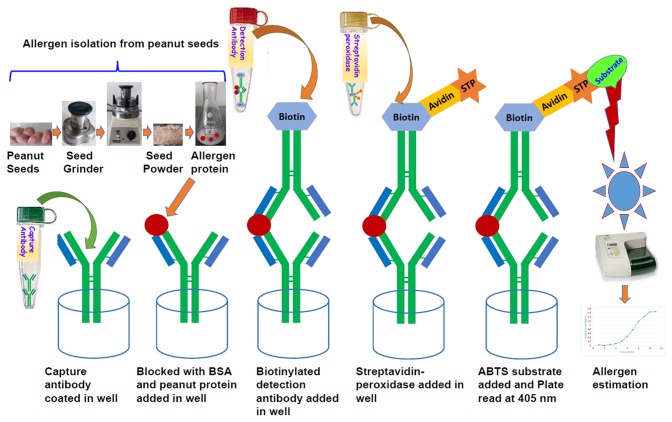
Systematic diagram showing the protocol for allergen estimation in peanut seed through sandwich ELISA.

The ELISA is sandwich format having double-antibody based on the specific interaction between antigen and antibody. The peanut allergen proteins are sandwich between two antibodies such as capture antibody and conjugated antibody with streptavidin peroxidase (Figure 1). For color development ABTS™ was used. The color intensity depends on the concentration of allergen protein present in the specific sample and measured with an iMark microplate reader (Bio-Rad) at 405 nm. The Microplate Manager (MPM) software was used while analyzing the optical density values of standards and samples. The systemic representation showed the key steps involved to develop the ELISA protocol, to estimate the peanut allergens through peanut seeds (Figure 1).

#### Grinding of Seeds, Homogenization and Purification

Sample extracts were prepared by grinding two grams of peanut seeds in fine powder and then dissolved in 40 ml of PBS-T (0.05% Tween in phosphate buffered saline, pH 7.4) containing 1M NaCl in 50 ml falcon tubes (Sarstedt No:55.476). After 2 h of gentle stirring on a rocking platform at room temperature, the aqueous phase was collected by centrifugation at 2,500 rpm at 4°C for 20 min. The aqueous phase was subsequently centrifuged at 3,500 rpm for 10 min at room temperature to remove residual traces and insoluble particles. Protein extracts were stored at −20°C until use.

#### Dilution Factor for Different Peanut Allergens

Dilution of a sample extract is critical for an ELISA which in turn determines the values of detection range for antibody and target antigen concentrations. Subsequently, the concentration of that specific allergens sample was estimated by multiplying the concentration found from the graph by the dilution factor (36, 37). By using different dilutions, we estimated the detection range of target antigen and antibody concentration. We standardized the dilution factors to detect each allergen proteins presents in the peanut seeds. Each sample was diluted in three different dilutions to detect the allergic protein present in seeds. The major allergen proteins such as Ara h 1 (1/1,000, 1/2,000, and 1/4,000), Ara h 2 (1/5,000, 1/10,000, and 1/20,000), Ara h 3 (1/5,000, 1/10,000, and 1/20,000), and Ara h 6 (1/40,000, 1/80,000, and 1/160,000) were diluted on a high range as compared to minor allergen protein, Ara h 8, which was diluted in low dilution range (1/10, 1/20, and 1/40). The optimal concentration of HRP-conjugated streptavidin was determined in the same way.

#### Steps Involved in ELISA

##### Antibody coating and blocking

Polystyrene microtiter plates (NUNC Maxisorp, Roskilde, Denmark) were coated with 100 μL mAb at 10 μL /10 ml in 50 mM carbonate buffer (100 μL/well). After overnight incubation at 4°C, the coated wells were washed three times with washing buffer (phosphate buffer containing 0.05% Tween 20) and left to block with 1% BSA for 30 min at room temperature followed by three times washing with washing buffer.

##### Capture of allergen samples and standard

The standard and samples were diluted in washing buffer containing 1% bovine serum albumin fraction V (Sigma Aldrich Cat No. 10735086001). The standard of each allergen was diluted to make 10 serial doubling dilutions in dilution buffer. Subsequently, the allergen samples (100 μL/well) with three different dilutions were added in respective wells and incubated at room temperature for 1 h.

##### Adding of detection antibody in plate

After incubation plates were washed three times and specific biotinylated anti Ara h mAb were diluted to 1/1,000 in washing buffer containing BSA (1 mg/mL) was added to the wells (100 μL/well) and incubated for 1 h at room temperature.

##### Streptavidin-enzyme conjugate

After three washes, HRP-conjugated Streptavidin diluted to 1/1,000 in washing buffer containing BSA (1 mg/mL) was added to the wells and incubated for 1 h at room temperature.

##### Addition of the substrate

The colorimetric substrate was added to the wells and which formed a colored solution when catalyzed by the enzyme. The wells were washed three times and 100 μL of 1 mM ABTS was added to each well. After 5 min, the color development was observed.

##### Detection through ELISA reader

The optical density (OD) was measured at 405 nm using BioRad Microplate Reader and the data were processed using Microplate Manager V 6.1 (Bio-Rad Laboratories).

### Spike and Recovery Studies

To test the accuracy of peanut allergens estimation, known amount of each allergen was spiked in the peanut extracts. The spike standard concentration for each allergen was ranging from 100 to 1,600 ng/ml for Ara h 1, 25 to 200 ng/ml for Ara h 2, and 6.25 to 100 ng/ml for Ara h 3, 10 to 160 for Ara h 3, and from 10 to 100 ng/ml for Ara h 8. These known amounts of individual standard (Ara h 1, Ara 2, Ara 3 Ara h 6, and Ara h 8) allergens were spiked in peanut extract and later calculating their final content in extract. The recovery was calculated as (B–C)/(A × 100), Where A = Known amount of peanut standard, B = Concentration of spiked standard, C = Concentration of peanut extract.

### Statistical Analysis and Sample Analysis Design

**Figure 2:**
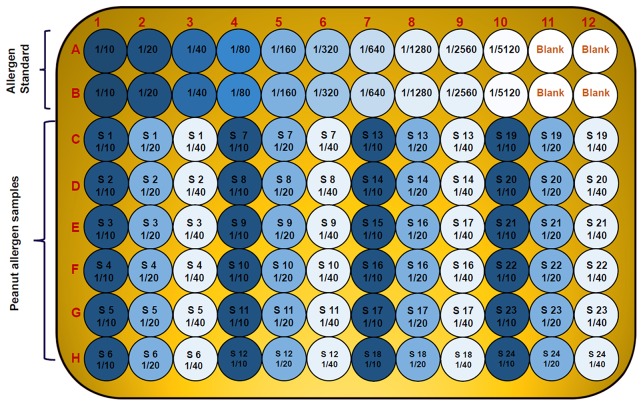
Diagram showing 96-well plate design and the serial dilution of allergen standard and peanut samples in three replicates and three dilutions. The wells A1 to A10 and B1 to B10 contain serial dilution of specific allergen standard; A11 to A12 and B11 to B12 are blank; while the C, D, E, F, G, and H wells are with unknown peanut samples in three different dilutions for specific allergen.

All experiments were conducted with three replications based on dilution factor (DF). Mean ELISA plate reading values (Optical Density; OD) for each standard and sample were used to plot a standard curve by placing each allergen standard concentration values on Y axis and respective OD values on X axis on an Excel spreadsheet on the computer. Using regression equation values, we estimated specific allergen concentration in each sample. Data are expressed as means ± standard deviation (SD). Allergen standards and samples were placed in the 96- well format plate as given in Figure 2. The statistical analysis of data was performed using SigmaPlot 11.0.

## Results

### Optimization of Dilution Factors (DFs) for Different Allergens From Peanut Seeds

**Figure 3:**
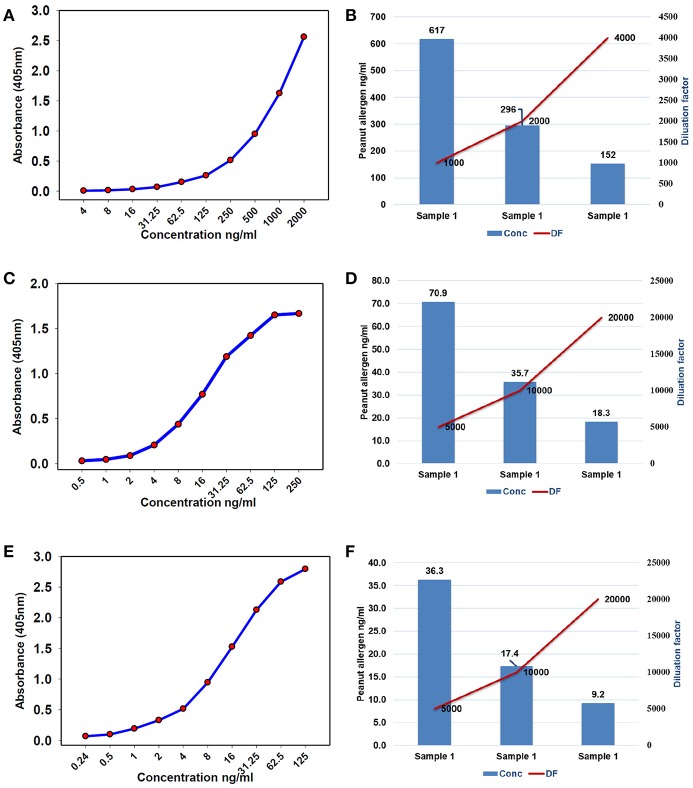
Standard curve and optimized dilutions for Ara h 1, Ara h 2, and Ara h 3 allergens in peanut seeds. **(A)** standard curve for Ara h 1 allergen and **(B)** standardization of dilution factor to estimate Ara h 1. **(C)** standard curve for Ara h 2 allergen and **(D)** standardization of dilution factor to estimate Ara h 2. **(E)** standard curve for Ara h 3 allergen, and **(F)** standardization of dilution factor to estimate Ara h 3.

**Figure 4:**
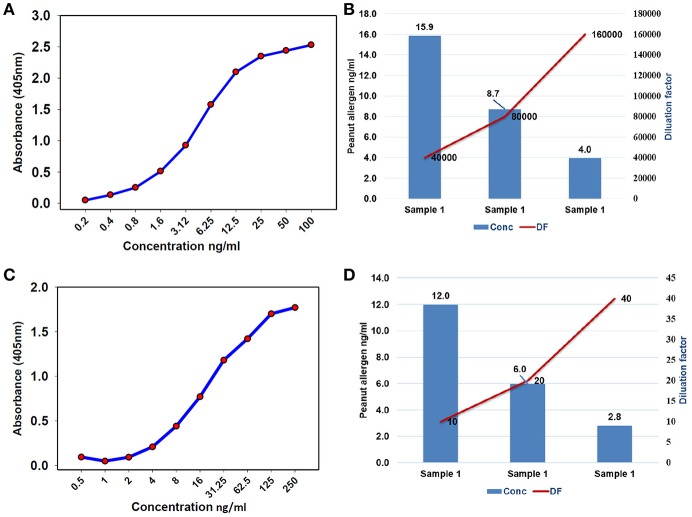
Standard curve and optimized dilutions for Ara h 6 and Ara h 8 allergens in peanut seeds. **(A)** standard curve for Ara h 6 allergen by sandwich ELISA, and **(B)** standardization of dilution factor to estimate Ara h 6. **(C)** standard curve for Ara h 8 allergen by sandwich ELISA, and **(D)** standardization of dilution factor to estimate Ara h 8.

To find the concentration of the different types of unknown allergens in the peanut seed samples, dilutions were performed on the different scale for specific allergens so that the relative concentrations fall according to their standard graph. Finally, after the absorbance of the specific allergens was determined, the concentrations of these specific allergens were determined by finding the location on the graph that corresponds to the absorbance of the standard. The concentrations of the allergens sample were estimated by multiplying samples concentration with dilution factor. For Ara h 1, the standard ranges from 0.004 to 2 μg/mL (Figure 3A), while it ranged from 0.0005 to 0.25 μg/mL and 0.00024 to 0.125 μg/mL for Ara h 2 and Ara h 3 (Figures 3C,E), respectively. The standard graph range of Ara h 6 and Ara h 8 was showing range between 0.0002 to 0.10 μg/mL and 0.0005 to 0.250 μg/mL (Figures 4A,C).

Based on the standard graph, the dilution factor was optimized on the basis of OD value and dilution of the peanut samples. Each allergen protein was estimated at different dilution factors (DF) in same samples. Here we used a number of dilutions in the peanut samples to detect the specific allergic protein in seeds. The Ara h 1 was detected on three serial doubling dilutions, 1:1,000, 1:2,000, and 1:4,000 (Figure 3B) while Ara h 2 and Ara h 3 detected on same dilution 1: 5,000, 1:10,000, and 1: 20,000 (Figures 3D,F). In peanut seeds, the Ara h 6 was detected in the high range (1:40,000, 1:80,000, and 1:160,000) dilution factor (Figure 4B), while Ara h 8 detected in a low range of dilution i.e., 1:10, 1:20, and 1:40 (Figure 4D). The result showed that in 1,000 dilution, the amount of Ara h 1 were detected 617 ng/ml while in 4000 dilution it decreased upto 152 ng/ml (Figures 3B). Likewise other peanut allergens Ara h 2, Ara h 3, Ara h 6, and Ara h 8 also showing similar pattern in their optimized result. The optimized dilution graphs (Figures 3D,F; 4B,D) showing that when we increasing the dilution, peanut allergen concentration is decreasing accordingly.

### Allergens Estimation in Peanut Seeds

The specificity of the sandwich ELISA was investigated by testing sample extracts of 38 peanut genotypes using different dilution factors for different allergens. Here, we used a single sample with three different dilutions and estimated four major (Ara h 1, Ara h 2, Ara h 3, and Ara h 6) and one minor (Ara h 8) allergen proteins in peanut seeds using specific antibodies. Supplementary Table 1 showed the selected genotypes on the basis of minimum and the maximum amount of allergens present in specific genotypes. The minimum and maximum amount of Ara h 1 was detected in ICG 311 (12.5 μg/g) and ICG 15380 (42733.87 μg/g) genotypes. The low and high level of Ara h 2 was detected in ICG 532 (22.2 μg/g) and ICG 14482 (20600.3 μg/g) while ICG 311 (157.8 μg/g) and ICG 12879 (14556.4 μg/g) showing low and high amount of Ara h 3, respectively. Genotype ICG 3992 (5767.6 μg/g) contained low amount of allergen while ICGV 91116 (42317.6 μg/g) contained high amount of Ara h 6 allergen among the genotypes. Ara h 8 showed low range of allergen ranging from lowest in ICG 311(0.084 μg/g) to highest in ICG 14705 (6.8 μg/g).

### Repeatability and Reproducibility

**Figure 5:**
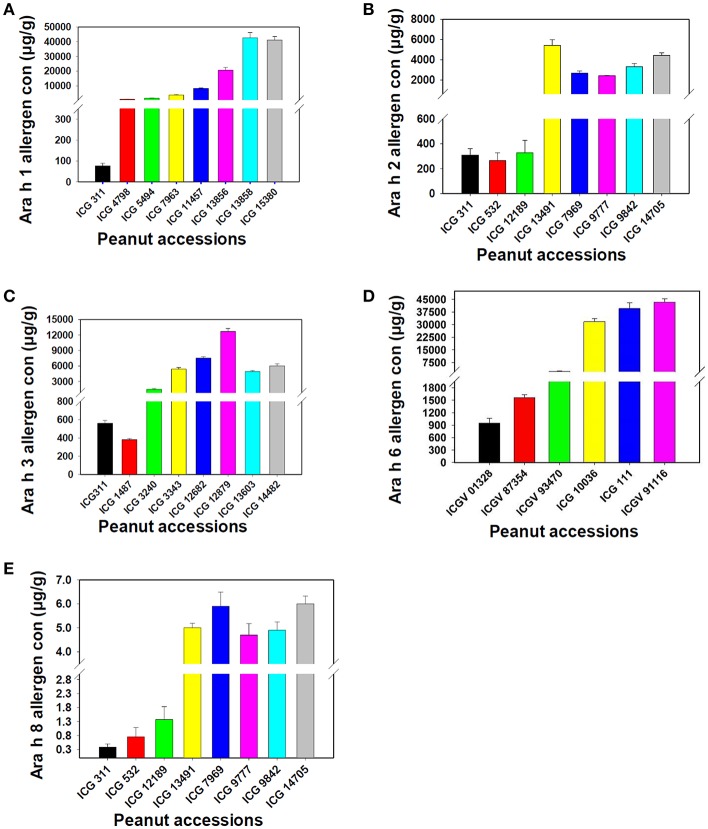
Assessment of the new protocol for its reproducibility for Ara h 1, Ara h 2, Ara h 3, Ara h 6 and Ara h 8 allergen proteins. **(A)** Ara h 1 allergen proteins, **(B)** Ara h 2 allergen proteins, **(C)**: Ara h 3 allergen proteins, **(D)**: Ara h 6 allergen proteins and **(E**) Ara h 8 allergen protein. The figure shows variation in the presence of allergen protein in low to high range in different peanut genotypes.

To validate the protocol, we repeated our experiments in three technical replicates with three dilutions optimized for each specific allergen. The assay had a linearity of *r*^2^ ranges from 0.987 to 0.999 for all the specific allergen concentration present in peanut seeds. The coefficient of variation (CV) of each peanut sample in triplicates was consistently <20% (Table 1). Assay repeatability and reproducibility was <15% CV as measured by using the three peanut samples (Table 1). We found that dilution factor and CV are the key features of this protocol. This method is fairly simple to estimate specific allergen protein present in a single peanut seed. The validation experiments showed linearity of the method and each specific allergen showing different range in different peanut seeds. By using this protocol, we identified wide range of specific allergens, Ara h 1 (77–42,609 μg/g) (Figure 5A, Table 1), Ara h 2 (265–5,426 μg/g) (Figure 5B, Table 1), Ara h 3 (382–12,676 μg/g) (Figure 5C, Table 1), Ara h 6 (949–43,375 μg/g) (Figure 5D, Table 1), and Ara h 8 (0.385–6 μg/g) (Figure 5E, Table 1) in peanut genotypes.

**Table 1:** The repeatability and precision of the ELISA-based assay in peanut seeds.

**S. No**	**Peanut genotype**	**Range in peanut seeds (μg/g)**	**Mean (μg/g)**	**Standard deviation (SD)**	**Range of (CV%)**	**Mean of (CV%)**
**Ara h 1 ALLERGEN PROTEIN**						
1	ICG 311	66–89	77	12	3–5	4
2	ICG 4798	719–936	842	112	5–15	9
3	ICG 5494	1,638–1,689	1,652	23	2–4	3
4	ICG 7963	3,496–4,105	3,761	312	2–11	7
5	ICG 11457	7,493–8,589	8,073	551	3–7	5
6	ICG 13856	18,253–22,080	20,481	1,989	1–10	5
7	ICG 13858	39,058–46,106	42,609	3,524	2–7	14
8	ICG 15380	39,426–43,859	41,008	2,474	1–15	7
**Ara h 2 ALLERGEN PROTEIN**						
9	ICG 311	253–361	307	54	22–25	24
10	ICG 532	208–331	265	62	12–19	16
11	ICG 12189	230–429	327	100	6–19	13
12	ICG 13491	5,005–6,062	5,426	561	5–17	10
13	ICG 7969	2,488–2,925	2,666	230	9–18	14
14	ICG 9777	2,390–2,467	2,422	41	1–15	7
15	ICG 9842	2,973–3,564	3,321	309	10–18	14
16	ICG 14705	4,136–4,642	4,432	264	4–17	11
**Ara h 3 ALLERGEN PROTEIN**						
17	ICG 311	406–646	559	32	8–24	8
18	ICG 1487	369–392	382	12	6–9	8
19	ICG 3240	1,274–1,626	1,452	176	9–22	15
20	ICG 3343	5,044–5,679	5,385	320	0.15–19	11
21	ICG 12682	7,161–7,758	7,524	250	2–29	13
22	ICG 12879	12,029–13,195	12,676	593	8–24	17
23	ICG 13603	4,707–5,125	4,909	209	7–12	9
24	ICG 14482	5,731–6,491	6,009	419	7–23	14
**Ara h 6 ALLERGEN PROTEIN**						
25	ICGV 01328	829–1,065	949	118	0.05–14	5
26	ICGV 87354	1,578–1,629	1,561	77	7–23	14
27	ICGV 93470	2,066–2,412	2,184	197	9–24	14
28	ICG 111	38,174–44,065	39,587	3,453	0.05–14	5
29	ICG 10036	29,711–32,982	31,731	1,809	4–15	10
30	ICGV 91116	41,288–45,998	43,375	1,947	5–11	8
**Ara h 8 ALLERGEN PROTEIN**						
31	ICG 311	0.275–0.503	0.385	0.114	3–17	11
32	ICG 532	0.415–1	0.752	0.332	4–20	13
33	ICG 12189	0.956–1.302	1.374	0.458	7–12	10
34	ICG 13491	4.8–5.2	5	0.189	5–22	16
35	ICG 7969	5.2–6.3	5.9	0.585	8–23	17
36	ICG 9449	4.2–5.2	4.7	0.477	17–24	21
37	ICG 9842	4.7–5.3	4.9	0.347	5–21	15
38	ICG 14705	5.6–6	6	0.322	11–27	20

*The range and mean of different allergen in selected Peanut genotypes. Data represents the mean of three replication ± SD and CV%*.

The accuracy of the developed method was determined by performing the spike and recovery test. The mean recoveries (*n* = 3) for all the five allergens, individually tested, were in the range of 81–115% (Table 2). This indicates that the developed method is accurate for all five peanut allergens through peanut extract.

**Table 2:** Recovery of five major allergens (Ara h 1, Ara h 2, Ara h 3, Ara h 6, and Ara h 8) from artificial spiked peanut allergens through ELISA in peanut seeds.

**S. No**.	**Concentration used for spiking in samples (ng/ml)**	**Detected concentration of spiked samples (ng/ml)**	**% recovery of allergen in spiked samples**	**CV (%)**
**RECOVERY OF SPIKED Ara h 1 FROM SAMPLES**				
1	100	105 ± 9	105.3	21.6
2	200	206 ± 8	103.0	22.2
3	400	376 ± 7	94.0	6.3
4	800	786 ± 10.9	98.3	17.6
5	1,600	1562 ± 11	97.6	11.4
**RECOVERY OF SPIKED Ara h 2 FROM SAMPLES**				
1	25	27 ± 3.2	106.8	10.9
2	50	57.9 ± 4.8	115.8	17.8
3	100	104.3 ± 0.3	104.4	12.8
4	150	148.7 ± 10.9	99.2	16.5
5	200	188.6 ± 7.6	94.3	20.7
**RECOVERY OF SPIKED Ara h 3 FROM SAMPLES**				
1	6.25	5.7 ± 0.77	91.4	9.9
2	12.5	12.17 ± 5.9	97.4	19.0
3	25	20.34 ± 3.24	81.4	7.0
4	50	53.45 ± 5.25	106.9	10.1
5	100	108.96 ± 3.8	109.0	9.4
**RECOVERY OF SPIKED Ara h 6 FROM SAMPLES**				
1	10	9.70 ± 2.9	97.0	15.5
2	20	17.94 ± 0.25	89.7	10.4
3	40	40.04 ± 4.31	100.1	14.8
4	80	83.97 ± 1.8	105.0	9.9
5	160	157.88 ± 4.37	98.6	8.9
**RECOVERY OF SPIKED Ara h 8 FROM SAMPLES**				
1	1.25	1.26 ± 0.15	100.78	5.26
2	2.5	2.48 ± 0.05	99.26	20.31
3	5	4.93 ± 0.36	98.53	16.16
4	10	53.45 ± 5.25	105.62	11.58
5	20	108.96 ± 3.8	100.56	12.30

Each sample was spiked with known amount of peanut allergens in peanut extract. Data represents the mean of three replicates ±SD and CV%. Formula used for detection (% recovery) = (B–C)/A^*^100. Where A, Known amount of peanut standard; B, Concentration of spiked standard; C, Concentration of peanut extract.

## Discussion

Peanut allergy is one of the major food allergies and leads to death in case of severe allergenic reaction. In order to tackle this problem, efforts toward the development of peanut varieties with minimum or nil allergens needs to be initiated in addition to efforts for developing cure for the allergy caused due to peanut consumption. Peanut genetic improvement program for this particular trait require a reliable, robust and cost-effective assay to screen the large scale germplasm and breeding material for major allergens. In this context, this article reports development of a cost-effective and reliable ELISA-based protocol to estimate five major allergens (Ara h 1, Ara h 2, Ara h 3, Ara h 6, and Ara h 8) in peanut seeds.

In serological tests, two-fold differences in measurements of replicates of the same sample are considered as acceptable (38). Current studies describe the standardization of dilution factor for peanut seed sample to analyse the specific allergen protein concentration through ELISA method. It has been observed that the concentration and dilution factors are inverse means and the concentration of the allergens decreased with the increasing dilution of the samples. All major allergen proteins (Ara h 1, Ara h 2, Ara h 3, Ara h 6, and Ara h 8) concentration was gradually decreased while increasing the dilution. For instance, the concentration of Ara h 1 was 617 ng/mL in 1,000 dilution, and in 2,000 dilution the concentration of Ara h 1 was detected as 296 ng/mL while in 4,000 dilution the concentration of Ara h 1 was detected as 152 ng/mL (Figure 3B). On the basis of optimized dilution factors, major allergen proteins were estimated in peanut seeds with increased precision. The results from our study show the importance of standard curve and dilution as an important parameter for quantifying the ELISA results (39). Dilution factor is an important step in ELISA before analyzing the results. Dilution assay apprises the actual concentration would be taken to experiment. To attained highly sensitive results for protein concentration, optimum values for dilution assay would provide a valid conclusion (39, 40). Earlier few attempts were made to quantify the levels of allergens in peanut seed using immuno assays and chromatography methods. But majority of them were expressed only in terms of percentages out of total protein present in the raw peanut seed. In one of the earlier studies, it was concluded that quantification of a single protein is not possible from a sample mixture of allergenic and non-allergenic proteins (41). Later in another study, using human sera from patients with documented history of peanut allergy, allergens from different peanut introductions were quantified and expressed in terms of ELISA optical density readings (4). In a recent study, Ara h 3 was quantified and found that it is the most abundant protein followed by Ara h 1 and Ara h 2 (34). However, in our study, Ara h 1 found in larger quantities followed by Ara h 6, Ara h 2, Ara h 3, and Ara h 8. Extraction method and proper dilutions of extracts plays crucial role in the precise quantification of these allergen proteins. Here in our study, we have thoroughly studied series of dilutions for each allergen and their impact on the quantification of allergens. These results clearly indicate three different dilutions for each major peanut allergen are required for precise detection in peanut seeds. Further the suggested 96-well plate design can allow the analysis of 24 samples in three replicates.

The newly developed ELISA protocol was validated on assay linearity, accuracy, repeatability and reproducibility. On the basis of primary results, few peanut genotypes were selected with low, medium and higher range of allergen proteins present in seeds. Our results indicated a different level of specific allergen profiles among peanut genotypes. Marked differences in specific peanut allergen profile were observed in peanut flour and peanut-based products such as peanut butter and other confectionary preparations for clinical use (42). In the serological assay, the variability produces continuous value were summarized by the coefficient of variation (CV), often reported in percentage (42, 43). The low CV observed in the current study while preparing dilutions provided more confidence in results produced for multiple genotypes using this protocol. Therefore, this protocol can provide accurate estimation for targeted five allergens and hence, can be very useful in accelerating the peanut research leading to detection and development of hypo-allergic peanuts.

The sample value obtained from an ELISA is dependent upon the interaction between the protein of interest and the ELISA's antibodies, and comparison of this interaction relative to a recombinant protein standard curve. We have validated this newly developed ELISA protocol for estimation of the major peanut allergens in peanut seeds by conducting the recovery experiments. The validation of ELISA protocol depends upon the recovery experiment which allow for a well-ordered presentation of the results (42).

## Conclusions

This study reports successful development and validation of an accurate ELISA method for allergen estimation in peanut seeds. The method developed here is a simple, fast and cost-effective method and can be applied to a large number of samples for specific allergen estimation. Our results clearly indicates three different dilutions for each major peanut allergen are required for precise detection in peanut seeds. Further the suggested 96-well plate design can allow the analysis of 24 samples in three replicates. This protocol can be very useful in accelerating the research in identifying peanut genotypes with minimum allergen proteins. Furthermore, the high level of phenotypic variation in a selected set of peanut germplasm showed very positive indication and hope toward efforts of developing allergen-free peanut genotypes in coming years using genomics-assisted breeding including genome editing.

## Author Contributions

MP conceived the idea. AP performed the experiment. MP, HS, RV, and AP designed the experiments, analyzed the data, and wrote the manuscript.

### Conflict of Interest Statement

The authors declare that the research was conducted in the absence of any commercial or financial relationships that could be construed as a potential conflict of interest.
