# Supplementary material for: An Improved Enzyme-Linked Immunosorbent Assay (ELISA) Based Protocol Using Seeds for Detection of Five Major Peanut Allergens Ara h 1, Ara h 2, Ara h 3, Ara h 6, and Ara h 8
Source: Front Nutr. 2019 Jun 5;6:68. doi: 10.3389/fnut.2019.00068 (PMC6560202; doi:10.3389/fnut.2019.00068)
Supplement: Supplementary file 1 [file Table_1.docx]

**Supplementary Table 1:** **Major allergens profiling in peanut seeds.** Data shows that Ara h 1, Ara h 2, Ara h 3 and Ara h 6 were major allergic proteins while Ara h 8 was the minor allergic protein.

| **S. No.** | **Peanut Accession** | **Ara h 1** | | **Ara h 2** | | **Ara h 3** |  | **Ara h 6** |  | **Ara h 8** | |
| --- | --- | --- | --- | --- | --- | --- | --- | --- | --- | --- | --- |
|  |  | **µg/g** | **CV%** | **µg/g** | **CV%** | **µg/g** | **CV%** | **µg/g** | **CV%** | **µg/g** | **CV%** |
| 1 | ICG 36 | 72 | 7 | 6977 | 13 | 776 | 5 | 13396 | 10 | 0 | 8 |
| 2 | ICG 4798 | 757 | 7 | 1158 | 5 | 753 | 9 | 17740 | 6 | 0 | 5 |
| 3 | ICG 5494 | 1629 | 3 | 2652 | 19 | 324 | 1 | 17116 | 11 | 1 | 25 |
| 4 | ICG 7963 | 3561 | 8 | 38196 | 12 | 12569 | 25 | 18291 | 15 | 1 | 13 |
| 5 | ICG 11457 | 7800 | 12 | 3368 | 10 | 10527 | 8 | 10751 | 4 | 0 | 13 |
| 6 | ICG 13856 | 18955 | 10 | 4515 | 29 | 14394 | 5 | 73948 | 47 | 2 | 4 |
| 7 | ICG 13858 | 38606 | 2 | 6074 | 1 | 14556 | 20 | 41224 | 20 | 1 | 9 |
| 8 | ICG 15380 | 42734 | 6 | 6967 | 1 | 6962 | 2 | 5886 | 25 | 3 | 17 |
| 9 | ICG 311 | 13 | 10 | 263 | 14 | 158 | 9 | 12722 | 8 | 0 | 24 |
| 10 | ICG 532 | 928 | 21 | 222 | 15 | 891 | 1 | 6437 | 14 | 0 | 17 |
| 11 | ICG 7969 | 707 | 11 | 2351 | 16 | 841 | 13 | 10169 | 12 | 3 | 18 |
| 12 | ICG 9777 | 792 | 14 | 2187 | 6 | 15481 | 24 | 29166 | 22 | 5 | 24 |
| 13 | ICG 9842 | 464 | 12 | 2662 | 4 | 10238 | 12 | 33779 | 16 | 3 | 19 |
| 14 | ICG 12189 | 2538 | 41 | 271 | 6 | 4506 | 0 | 11858 | 1 | 3 | 11 |
| 15 | ICG 13491 | 1318 | 12 | 5241 | 1 | 12246 | 21 | 6212 | 6 | 2 | 16 |
| 16 | ICG 14705 | 12112 | 2 | 3862 | 24 | 8971 | 0 | 11076 | 20 | 2 | 18 |
| 17 | ICG 311 | 13 | 10 | 263 | 14 | 158 | 9 | 12722 | 8 | 0 | 24 |
| 18 | ICG 1487 | 45 | 7 | 1524 | 15 | 318 | 0 | 13662 | 8 | 3 | 9 |
| 19 | ICG 3240 | 460 | 4 | 6000 | 13 | 1326 | 13 | 15027 | 17 | 4 | 6 |
| 20 | ICG 3343 | 715 | 4 | 4740 | 1 | 4232 | 4 | 11225 | 6 | 4 | 6 |
| 21 | ICG 12682 | 1368 | 6 | 6785 | 9 | 8961 | 12 | 10005 | 6 | 5 | 16 |
| 22 | ICG 12879 | 2443 | 11 | 9493 | 26 | 11791 | 11 | 6644 | 1 | 3 | 20 |
| 23 | ICG 13603 | 566 | 17 | 7936 | 2 | 5306 | 20 | 6233 | 0 | 2 | 6 |
| 24 | ICG 14482 | 4540 | 6 | 20600 | 9 | 7195 | 7 | 5487 | 5 | 2 | 13 |
| 25 | ICGV01328 | 2968 | 11 | 5114 | 1 | 5363 | 6 | 829 | 7 | 0.55 | 23 |
| 26 | ICG 875 | 382 | 13 | 7187 | 15 | 4330 | 6 | 13359 | 6 | 0 | 20 |
| 27 | ICG 3992 | 3222 | 10 | 5187 | 1 | 8837 | 9 | 57168 | 5 | 7 | 13 |
| 28 | ICG 8106 | 1119 | 13 | 84434 | 42 | 3261 | 14 | 14330 | 4 | 5 | 5 |
| 29 | ICG 10036 | 962 | 18 | 6717 | 2 | 1009 | 8 | 29101 | 10 | 6 | 19 |
| 30 | ICG 91116 | 15268 | 10 | 4988 | 26 | 5004 | 10 | 42318 | 9 | 5 | 24 |
| 31 | ICG 297 | 55 | 11 | 5580 | 8 | 14239 | 30 | 8300 | 4 | 0 | 5 |
| 32 | ICG 311 | 13 | 10 | 263 | 14 | 158 | 9 | 12722 | 8 | 0 | 24 |
| 33 | ICG 1703 | 332 | 21 | 5739 | 10 | 10296 | 1 | 21980 | 8 | 3 | 17 |
| 34 | ICG 2286 | 847 | 16 | 9060 | 4 | 7103 | 11 | 27220 | 3 | 3 | 9 |
| 35 | ICG 2106 | 608 | 23 | 5990 | 3 | 5198 | 10 | 16579 | 7 | 4 | 7 |
| 36 | ICG 2772 | 603 | 8 | 5639 | 12 | 9415 | 15 | 18016 | 13 | 5 | 16 |
| 37 | ICG 3673 | 1333 | 7 | 3357 | 1 | 278 | 19 | 25545 | 23 | 7 | 4 |
| 38 | ICG 5236 | 1536 | 10 | 9738 | 45 | 220 | 56 | 27698 | 19 | 7 | 4 |
